# The prognostic and predictive value of mRNA expression of vascular endothelial growth factor family members in breast cancer: a study in primary tumors of high-risk early breast cancer patients participating in a randomized Hellenic Cooperative Oncology Group trial

**DOI:** 10.1186/bcr3354

**Published:** 2012-11-12

**Authors:** Helena Linardou, Konstantine T Kalogeras, Ralf Kronenwett, George Kouvatseas, Ralph M Wirtz, Flora Zagouri, Helen Gogas, Christos Christodoulou, Angelos K Koutras, Epaminondas Samantas, Dimitrios Pectasides, Dimitrios Bafaloukos, George Fountzilas

**Affiliations:** 1 First Department of Medical Oncology, "Metropolitan" Hospital, Eth. Makariou 9 & El. Venizelou 1, Athens, 18547, Greece; 2 Department of Medical Oncology, "Papageorgiou" Hospital, Aristotle University of Thessaloniki School of Medicine, Ring Road of Thessaloniki, Nea Efkarpia, Thessaloniki, 56429, Greece; 3 Translational Research Section, Hellenic Cooperative Oncology Group, Data Office, Hatzikonstanti 18, Athens, 11524, Greece; 4 Siemens Healthcare Diagnostics, Nattermann-Allee 1, Cologne, 50829, Germany; 5 Health Data Specialists, Ltd, Krimeas 2, Athens, 11526, Greece; 6 Oncology Section, Department of Clinical Therapeutics, "Alexandra" Hospital, University of Athens School of Medicine, Vas. Sofias 80, Athens, 11528, Greece; 7 First Department of Medicine, "Laiko" General Hospital, University of Athens School of Medicine, Ag. Thoma 17, Athens, 11527, Greece; 8 Second Department of Medical Oncology, Metropolitan Hospital, Eth. Makariou 9 & El. Venizelou 1, Athens, 18547, Greece; 9 Division of Oncology, Department of Medicine, University Hospital, University of Patras Medical School, Rio, Patras, 26504, Greece; 10 Third Department of Medical Oncology, "Agii Anargiri" Cancer Hospital, Kalyftaki, Nea Kifissia, Athens, 14564, Greece; 11 Oncology Section, Second Department of Internal Medicine, "Hippokration" Hospital, University of Athens School of Medicine, Vas. Sofias 114, Athens, 11527, Greece

## Abstract

**Introduction:**

The main prognostic variables in early breast cancer are tumor size, histological grade, estrogen receptor/progesterone receptor (ER/PgR) status, number of positive nodes and human epidermal growth factor receptor 2 (HER2) status. The present study evaluated the prognostic and/or predictive value of vascular endothelial growth factor (VEGF) family members in high-risk early breast cancer patients treated with adjuvant chemo-hormonotherapy.

**Methods:**

RNA was isolated from 308 formalin-fixed paraffin-embedded primary tumor samples from breast cancer patients enrolled in the HE10/97 trial, evaluating adjuvant dose-dense sequential chemotherapy with epirubicin followed by cyclophosphamide, methotrexate, fluorouracil (CMF) with or without paclitaxel (E-T-CMF versus E-CMF). A fully automated method based on magnetic beads was applied for RNA extraction, followed by one-step quantitative RT-PCR for mRNA analysis of VEGF-A, -B, -C and vascular endothelial growth factor receptor (VEGFR) 1, 2, 3.

**Results:**

With a median follow-up of 8 years, 109 patients (35%) developed a relapse and 80 patients (26%) died. In high VEGF-C and VEGFR1 mRNA expressing tumors, ER/PgR-negative tumors (Fisher's exact test, *P *= 0.001 and *P *= 0.021, respectively) and HER2-positive tumors (*P *<0.001 and *P *= 0.028, respectively) were more frequent than in low VEGF-C and VEGFR1 expressing tumors, respectively. From the VEGF family members evaluated, high VEGFR1 mRNA expression (above the 75^th ^percentile) emerged as a significant negative prognostic factor for overall survival (OS; hazard ratio (HR) = 1.60, 95% confidence interval (CI): 1.01 to 2.55, Wald's *P *= 0.047) and disease-free survival (DFS; HR = 1.67, 95% CI: 1.13 to 2.48, *P *= 0.010), when adjusting for treatment group. High VEGF-C mRNA expression was predictive for benefit from adjuvant treatment with paclitaxel (E-T-CMF arm) for OS (test for interaction, Wald's *P *= 0.038), while in multivariate analysis the interaction of VEGF-C with taxane treatment was significant for both OS (Wald's *P *= 0.019) and DFS (*P *= 0.041) and continuous VEGF-B mRNA expression values for OS (*P *= 0.019).

**Conclusions:**

The present study reports, for the first time, that VEGF-C mRNA overexpression, as assessed by qRT-PCR, has a strong predictive value in high-risk early breast cancer patients undergoing adjuvant paclitaxel-containing treatment. Further studies are warranted to validate the prognostic and/or predictive value of VEGF-B, VEGF-C and VEGFR1 in patients treated with adjuvant therapies and to reveal which members of the VEGF family could possibly be useful markers in identifying patients who will benefit most from anti-VEGF strategies.

**Trial registration:**

Australian New Zealand Clinical Trials Registry (ANZCTR) ACTRN12611000506998

## Introduction

The main prognostic variables in early breast cancer are tumor size, grade, estrogen and progesterone receptor (ER/PgR) status, number of positive nodes and human epidermal growth factor receptor 2 (HER2) status [1]. These and other clinicopathological parameters are commonly utilized to identify patients who are more likely to benefit from adjuvant chemotherapy and hormonal therapy. However, a large number of other molecules are being extensively investigated for their predictive and prognostic value, since most existing clinicopathological models have only moderate predictive power and do not account for the molecular diversity of tumors [2].

Recent experimental and clinical studies have suggested the essential role of angiogenesis in breast cancer among many other tumor types. The members of the vascular endothelial growth factor (VEGF) family and their receptors (VEGFRs) have a central function in angiogenesis and the formation of vascular networks. Today, we recognize five VEGFs (VEGF-A, -B, -C, -D, -E), with the first three being better characterized. VEGF-A and -B are considered mainly angiogenic, while VEGF-C is thought to be more lymphangiogenic. Their binding partners are three different tyrosine kinase receptors, VEGFR1 (or Flt-1), VEGFR2 (or KDR/Flk-1) and VEGFR3 (or Flt-4) [3,4]. VEGF-A is expressed at low levels in normal adult life and is over-expressed during wound healing and tissue regeneration. It has two known receptors, VEGFR1 and 2, mainly expressed in endothelial cells. VEGF-B is expressed at higher levels in cardiac and skeletal muscle cells, it forms heterodimers with VEGF-A and has two known binding receptors, VEGFR1 and neuropilin-1. VEGF-C was initially identified as a ligand for the tyrosine kinase receptor VEGFR3, which is associated with the lymphatic vasculature [5]. VEGF-C is also a ligand for VEGFR2, which it shares with VEGF-A and -D. A number of recent studies have investigated the role of VEGF-C in human tumors [6]; however, few have explored its role in human breast cancer. In those, VEGF-C has been proposed to be an inducer of tumor lymphangiogenesis and, therefore, an important promoter of breast cancer metastasis [7-9].

Angiogenesis is of central importance in the growth and metastasis of tumors and in particular of breast cancer [10,11]. Both VEGF-A and -B, and their receptors, have been found to be expressed in several different tumor types, including breast cancer [12]. Recently, VEGF-A has emerged as an important factor for progression in many tumor types and has been the target of bevacizumab [13]. However, its specific role in cancer has not been fully elucidated as yet.

The prognostic and clinicopathological significance of VEGF-A in breast cancer, both in node positive and node negative patients [14,15], has been evaluated by ELISA assays in several studies, less frequently and with controversial results by immunohistochemistry [15] and even less frequently by modern RT-PCR assays [16-18]. The role of VEGF-B is even less studied and understood [19]. Tumor-induced lymphangiogenesis has only recently been described and remains largely unexplored. Recent studies have suggested that it is mainly driven by VEGF-A and VEGF-C [20]. Furthermore, there is very limited information regarding the predictive role of any of the VEGF family members in breast cancer patients undergoing systemic treatment, hormonal therapy and/or chemotherapy.

Although the expression of VEGF family members at the protein level is well studied, the relationship of VEGF family members mRNA expression with various parameters or tumor progression is unclear. Quantitative RT-PCR (qRT-PCR) is a powerful tool that allows the selective measurement of mRNA expression levels in cancer cells, offering accurate relative quantification of mRNA levels of specific biomarkers [21] in formalin-fixed paraffin-embedded (FFPE) tumor tissue samples [22].

We initiated this study, with the aim of evaluating the mRNA expression patterns of VEGF family members in high-risk early breast cancer patients who had participated in a large randomized adjuvant chemo-hormonotherapy trial. We utilized a one-step qRT-PCR technique and correlated VEGF family members' mRNA expression with well-characterized clinicopathological parameters. Last but not least, we sought to explore the prognostic/predictive significance of mRNA expression of the evaluated VEGF family members on disease-free survival (DFS) and overall survival (OS) in high-risk operable breast cancer patients.

## Materials and methods

### Patient population

Tumor tissue samples were retrospectively obtained from patients with high-risk operable breast cancer, who had participated in a prospective randomized phase III study of dose-dense sequential chemotherapy with epirubicin (E), followed by intensified CMF with or without paclitaxel (T, Taxol^®^, Bristol Myers-Squibb, Princeton, NJ, USA), by the Hellenic Cooperative Oncology Group (HE10/97). Due to the retrospective nature of the present translational research study, collection of FFPE primary tumor tissue samples was possible in 317 patients only, due to logistical/organizational barriers. The clinical study randomized a total of 595 high-risk (T_1-3_N_1_M_0 _or T_3_N_0_M_0_) breast cancer patients from 1997 to 2000, in order to explore the effect of dose-dense sequential chemotherapy with or without paclitaxel (E-T-CMF versus E-CMF), primarily on DFS and secondarily on OS. The trial was included in the Australian New Zealand Clinical Trials Registry (ANZCTR) and allocated Registration Number ACTRN12611000506998. Chemotherapy cycles were administered every two weeks and patients received granulocyte-colony stimulating factor (G-CSF) support. The present study was approved by the Bioethics Committee of the Aristotle University of Thessaloniki and patients provided written informed consent prior to enrollment. All participating patients also gave written informed consent for research use of their biological material. The results of the HE10/97 study have been previously reported [23].

Data collected for this retrospective experimental study included treatment arm, age, menopausal status, interval from operation, number of positive nodes, tumor size, histological grade and adjuvant radiotherapy/hormonotherapy. Primary tumor diameter and axillary nodal status were obtained from the pathology report. Histological grade was evaluated according to the Scarff, Bloom and Richardson system.

### Tissue microarray construction

Representative H & E stained sections from the tissue blocks were reviewed by a pathologist and the most representative tumor areas were marked for the construction of the tissue microarray (TMA) blocks, as previously described [24]. Each case was represented by two tissue cores, 1.5 mm in diameter, with each TMA block also containing cores from various neoplastic, non-neoplastic and reactive tissues serving as assay controls. Cases not represented, damaged or inadequate on the TMA sections were re-cut from the original blocks and these sections were used for protein and gene analysis.

### Immunohistochemistry

Immunohistochemistry (IHC) for ER (clone 6F11, Novocastra™, Leica Biosystems, Newcastle, UK), PgR (clone 1A6, Novocastra™, Leica Biosystems) and HER2 (A0485 polyclonal antibody, Dako, Glostrup, Denmark) was performed on serial 2.5 μm thick TMA sections, using a Bond Max™ autostainer (Leica Microsystems, Wetzlar, Germany), as previously described [24]. All cases were also stained for vimentin (clone V9, Dako) and cytokeratin 8/18 (clone 5D3, Novocastra™, Leica Biosystems), which were used as control stains for tissue immunoreactivity and fixation, as well as identification of tumor cells. Tissue samples negative for the above antibodies were excluded from the study. The evaluation of all IHC sections was done by experienced breast cancer pathologists, blinded as to the patients' clinical characteristics and survival data.

### Interpretation of the immunohistochemistry results

ER, PgR and HER2 protein expression was evaluated according to established or proposed criteria [25,26]. The ER and PgR immunostaining was scored using the histoscore method. Tissue sections stained for ER/PgR were considered to be positive when ≥1% of the neoplastic cells displayed nuclear immunoreactivity [25]. HER2 protein expression was scored according to the recent guideline recommendations (scores 0 to 3+) [26]. HER2 was considered to be positive in cases with an IHC score of 3+ (uniform, intense membrane staining in >30% of the invasive tumor cells).

### Fluorescence *in situ *hybridization

TMA sections or whole tissue sections (5 μm thick) were used for fluorescence *in situ *hybridization (FISH) analysis, using the ZytoLight^® ^SPEC *HER2*/*TOP2A*/CEN17 triple color probe (ZytoVision, Bremerhaven, Germany), as previously described [27]. Four carcinoma cell lines (MDA-MB-231, MDA-MB-175, MDA-MB-453, and SK-BR-3) from the Oracle HER2 Control Slide (Leica Biosystems), with a known *HER2 *gene status, were also used as a control of the FISH assays and analyzed for *HER2 *genomic status. *TOP2A *gene amplification was not evaluated for the purposes of the present study.

For the evaluation of the *HER2 *gene status, non-overlapping nuclei from the invasive part of the tumor were randomly selected and scored. The virtual slides of HER2, ER or PgR stains were used for selecting the invasive part of the tumor in each TMA. The virtual slides were created as previously described [28]. Twenty tumor nuclei were counted according to Press *et al. *[29]. The *HER2 *gene was considered to be amplified when the ratio of the gene probe/centromere probe was ≥2.2 [26], or the *HER2 *copy number was >6 [30]. In cases with values at or near the cut-off (1.8 to 2.2), an additional 20 or 40 nuclei were counted and the ratio was recalculated. In cases with a borderline ratio at 60 nuclei, additional FISH assays were performed in whole sections. HER2 was considered to be positive if it was amplified (ratio ≥2.2 or copy number >6) by FISH and/or a HER2 score of 3+ was obtained by IHC.

### RNA isolation from formalin-fixed paraffin-embedded tissue and quantitative reverse transcription-polymerase chain reaction assessment

H & E sections from all available FFPE tissue specimens were evaluated histologically by a certified pathologist who recorded percentage of tumor cell content in each one. Prior to RNA isolation, macrodissection of tumor areas was performed in most of the FFPE sections with <50% tumor cell content. The tumor cell content was >30% in practically all (97%) of the samples and >50% in the majority (76%) of the samples. More than one FFPE section was used for RNA extraction when the tumor surface of a given sample was less than 0.25 cm^2^, in an effort to minimize the rate of technical failures in the RNA extraction.

Sufficient RNA was isolated from 308 FFPE specimens followed by qRT-PCR, as previously described [31]. From each FFPE section or macrodissected tissue fragment (10 μm thick), RNA was isolated using a standardized fully automated isolation method for total RNA from FFPE tissue, based on silica-coated magnetic beads (VERSANT Tissue Preparation Reagents, Siemens Healthcare Diagnostics, Tarrytown, NY, USA) in combination with a liquid handling robot, as previously described in detail [22]. The method involves extraction-integrated deparaffinization and DNase I digestion steps. DNA-free total RNA was eluted with 100 μL elution buffer and stored at -80°C.

One-step qRT-PCR was applied for the relative quantification of VEGF-A, VEGF-B, VEGF-C, VEGFR1, VEGFR2 and VEGFR3 mRNA expression, by using gene-specific TaqMan^® ^based assays. Forty cycles of nucleic acid amplification were applied and the cycle threshold (CT) values of the target genes were identified. CT values were normalized by subtracting the CT value of the housekeeping gene *RPL37A *(ribosomal protein L37a) from the CT value of the target genes (ΔCT). RNA results were then reported as 40-ΔCT values, which correlate proportionally with the mRNA expression level of the target genes. For assessment of DNA contamination, a qPCR analysis specific for the *PAEP *gene (progestagen-associated endometrial protein) was performed, without the preceding reverse-transcription step. Samples were considered to be substantially free of DNA when CT values above 38 were detected. In the case of DNA contamination, samples were manually re-digested with DNase I. The quantity of RNA following isolation (yield) was checked by measuring RPL37A expression as a surrogate marker for amplifiable mRNA. Samples with average RPL37A CT values <32 were considered to have sufficient RNA and were eligible for analysis. Only 3 of the 311 extracted samples (1%) had an average RPL37A CT value of ≥32 and were, therefore, excluded from further analysis, resulting in successful RNA extraction from 99% of the samples.

Expression of the target genes, as well as the reference gene *RPL37A*, was assessed in triplicate by qRT-PCR using the SuperScript III PLATINUM One-Step Quantitative RT-PCR System with ROX (Invitrogen, Karlsruhe, Germany) in an ABI PRISM 7900HT (Applied Biosystems, Darmstadt, Germany) [21]. The lengths of the amplicons detected by the VEGF-A, VEGF-B, VEGF-C, VEGFR1, VEGFR2, VEGFR3 and RPL37A assays were 80 bp, 81 bp, 77 bp, 85 bp, 68 bp, 70 bp and 65 bp, respectively, with PCR efficiencies [E = 1^(10-slope)^] of 85.5, 110.3, 88.2, 95.7, 94.3, 84.7 and 86.0%, respectively. A commercially available human reference RNA (Stratagene qPCR Human Reference Total RNA, Agilent Technologies, Waldbronn, Germany) was used as positive control. No-template controls were assessed in parallel to exclude contamination.

The Primer/Probe (FAM/TAMRA-labeled) sets used for amplification of the target and reference genes were the following (5' -> 3'):

VEGF-A Probe CACCATGCAGATTATGCGGATCAAACCT

Forward Primer GCCCACTGAGGAGTCCAACA

Reverse Primer TCCTATGTGCTGGCCTTGGT

VEGF-B Probe CACATCTATCCATGACACCACTTTCCTCTGG

Forward Primer TGGCAGGTAGCGCGAGTAT

Reverse Primer CCCTGTCTCCCAGCCTGAT

VEGF-C Probe TTGAGTCATCTCCAGCATCCGAGGAAA

Forward Primer CCACAGATGTCATGGAATCCAT

Reverse Primer TGCCTGGCTCAGGAAGATTT

VEGFR1 Probe TGCTGTCGCCCTGGTAGTCATCAAACA

Forward Primer CATGGGAGAGGCCAACAGA

Reverse Primer AACCTTTGAAGAACTTTTACCGAATG

VEGFR2 Probe TCTTGGCATCGCGAAAGTGTATCCACA

Forward Primer TTCCAAGTGGCTAAGGGCAT

Reverse Primer CGTGCCGCCAGGTCC

VEGFR3 Probe TGCCTGCTTCCCTGGGTAGTCCC

Forward Primer GCACCCACTTACCCCGC

Reverse Primer GAGTTTAACTCAGGTGTCACCTTTGA

RPL37A Probe TGGCTGGCGGTGCCTGGA

Forward Primer TGTGGTTCCTGCATGAAGACA

Reverse Primer GTGACAGCGGAAGTGGTATTGTAC

### Statistical analysis

For all VEGF family members the quartiles (first, median and third) were examined as possible thresholds for prognostic significance in terms of OS or DFS. If a cut-off showed prognostic significance it was used to dichotomize the tumors into low and high expressing tumors. Otherwise, only the normalized mRNA expression values were used in the analysis as a continuous variable to evaluate prognostic significance.

OS was measured from the date of randomization until death from any cause. Surviving patients were censored at the date of last contact. DFS was measured from the date of randomization until recurrence of tumor, secondary neoplasm or death from any cause [32]. Time-to-event distributions were estimated using Kaplan-Meier curves. Continuous variables were presented as medians with the corresponding range and categorical variables as frequencies with the respective percentages. Associations of ligands and receptors with basic patient and tumor characteristics were examined using the Fisher's exact test for categorical variables and the Mann-Whitney or the Kruskall-Wallis tests, where appropriate, for continuous variables.

Correlations between the VEGF family ligands and their associated receptors were calculated using the Spearman's rank correlation coefficient (Rho). Cox regression analyses were performed to assess the relationship between markers and OS or DFS. Interactions between markers and treatment group, as well as between ligands and their associated receptors were also explored in the Cox models. In the multivariate Cox regression analysis, a backward selection procedure with a removal criterion of *P *>0.10 based on the likelihood ratio test was performed to identify significant variables among the following: treatment group (E-CMF versus E-T-CMF), menopausal status (post versus pre), time interval from breast surgery operation (>4 weeks versus 2 to 4 weeks versus <2 weeks), histological grade (III-IV versus I-II), tumor size (>5cm versus 2 to 5cm versus ≤2cm), number of positive axillary nodes (≥4 versus 0 to 3), ER/PgR status (positive versus negative versus missing), HER2 status (negative versus positive versus missing), hormonal therapy (yes versus no), radiotherapy (yes versus no), VEGF-A (continuous mRNA values), VEGF-B (continuous mRNA values), VEGF-C (high versus low at the 75^th ^percentile), VEGFR1 (high versus low at the 75^th ^percentile), VEGFR2 (continuous mRNA values), VEGFR3 (continuous mRNA values).

The design of the study is prospective-retrospective as described in Simon *et al. *[33]. Results of this study are presented according to reporting recommendations for tumor marker prognostic studies [34]. The SPSS software was used for statistical analysis (SPSS for Windows, version 15.0, SPSS Inc.). No adjustment for multiple comparisons is reported.

## Results

### Patient and tumor characteristics

A total of 308 primary tumor tissue samples were analyzed as stated in the 'Methods' section. Basic clinical and pathological characteristics of the patients (Table 1) were well balanced according to adjuvant chemotherapy, except for histological grade (*P *= 0.008), in agreement with the corresponding results presented in the clinical paper [23]. In addition, there were no significant differences in important clinicopathological characteristics between the patients included in the present study and the rest of the HE10/97 randomized patients, for which tissue samples were not available.

**Table 1:** Basic patient and tumor characteristics.

	E-T-CMF		E-CMF		All patients	
Number	141		167		308	
Age (years)						
Median	50		50		50	
Range	24 to 76		22 to 78		22 to 78	
Number of nodes removed						
Median	19		20		20	
Range	5 to 59		4 to 53		4 to 59	
Number of positive nodes						
Median	7		6		6	
Range	0 to 54		0 to 49		0 to 54	
	**N**	**%**	**N**	**%**	**N**	**%**
0 to 3 nodes	30	21.3	45	26.9	75	24.4
≥4	111	78.7	122	73.1	233	75.6
Menopausal status						
Premenopausal	76	53.9	89	53.3	165	53.6
Postmenopausal	65	46.1	78	46.7	143	46.4
Type of operation						
Modified radical mastectomy	111	78.7	132	79.0	243	78.9
Breast conserving surgery	30	21.3	35	21.0	65	21.1
Interval from operation						
<2 weeks	17	12.1	24	14.4	41	13.3
2 to 4 weeks	72	51.1	70	41.9	142	46.1
>4 weeks	52	36.9	73	43.7	125	40.6
Tumor size						
≤2cm	40	28.4	52	31.1	92	29.9
2 to 5cm	79	56.0	83	49.7	162	52.6
>5cm	22	15.6	32	19.2	54	17.5
Histological grade^a^						
I-II	60	42.6	97	58.1	157	51.0
III-IV	81	57.4	70	41.9	151	49.0
ER/PgR status						
Negative	28	19.9	29	17.4	57	18.5
Positive	95	67.4	108	64.7	203	65.9
Missing data	18	12.8	30	18.0	48	15.6
HER2 status^b^						
Negative	79	56.0	100	59.9	179	58.1
Positive	35	24.8	32	19.2	67	21.8
Missing data	27	19.1	35	21.0	62	20.1
Adjuvant RT						
No	21	14.9	33	19.8	54	17.5
Yes	119	84.4	133	79.6	252	81.8
Missing data	1	0.7	1	0.6	2	0.6
Adjuvant HT						
No	8	5.7	18	10.8	26	8.4
Yes	133	94.3	149	89.2	282	91.6
Tamoxifen	120	85.1	127	76.0	247	80.2
LH-RH agonist	65	46.1	62	37.1	127	41.2
Aromatase inhibitors	5	3.5	6	3.6	11	3.6
Other	1	0.7	2	1.2	3	1.0

^a^The two treatment arms were not balanced in terms of histological grade (*P *= 0.008); ^b^positive HER2 status: HER2 3+ by IHC and/or *HER2 *amplification by FISH. CMF, cyclophosphamide, methotrexate, fluorouracil; ER, estrogen receptor; FISH, fluorescence *in situ *hybridization; HER2, human epidermal growth factor receptor 2; HT, hormonal therapy; IHC, immunohistochemistry; LH-RH, luteinizing hormone-releasing hormone; PgR, progesterone receptor; RT, radiation therapy.

The median follow-up period was eight years (range 7 to 126 months). A total of 109 patients developed a relapse (35%) and 80 patients died (26%). Median OS has not been reached yet, while median DFS was 121 months (95% CI: 105 to 138). The five-year OS rate was 83% (95% CI: 79 to 87) and the seven-year OS rate was 77% (95% CI: 72 to 81). The five-year DFS rate was 71% (95% CI: 66 to 76) and the seven-year DFS rate was 66% (95% CI: 60 to 71).

### Normalized mRNA expression

**Figure 1:**
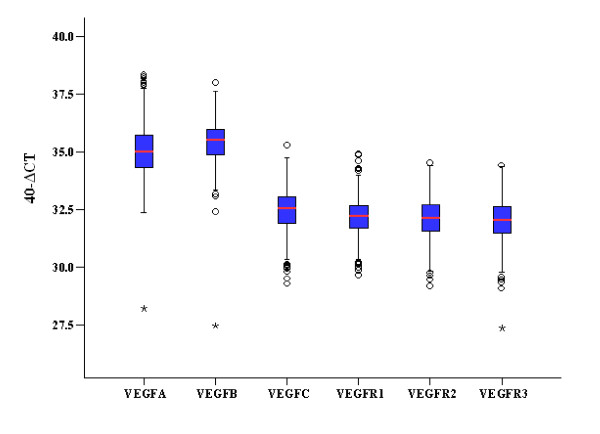
**Distribution of mRNA expression values**. Normalized mRNA expression values (40-delta CT) of all qRT-PCR evaluated VEGF family members are presented. CT, cycle threshold; VEGF, vascular endothelial growth factor.

The distribution of normalized mRNA expression (40-ΔCT values) of each VEGF family gene is shown in Figure 1. The median value for VEGF-A was 35.0 (range: 28.2 to 38.3), for VEGF-B 35.5 (range: 27.5 to 38.0), for VEGF-C 32.5 (range: 29.3 to 35.3), for VEGFR1 32.2 (range: 29.7 to 34.9), for VEGFR2 32.1 (range: 29.2 to 34.5) and for VEGFR3 32.0 (range: 27.4 to 34.4). All examined genes followed a unimodal distribution pattern.

Spearman's correlations between ligands and their associated receptors were examined. More specifically, there were statistically significant weak to moderate positive correlations between VEGF-A and receptors VEGFR1 and 2, VEGF-B and VEGFR1, as well as between VEGF-C and receptors VEGFR2 and 3 (Rho ranges from 0.30 to 0.56, *P *<0.001 in all cases), in agreement with the expected binding of the ligands.

### Associations of vascular endothelial growth factor family gene expression with patient and tumor characteristics

The mRNA expression of all VEGF family genes was evaluated for associations with the following patient and tumor characteristics: age, treatment group, menopausal status, ER/PgR status, HER2 status, number of positive nodes, tumor size, histological grade and adjuvant treatment (hormonal and radiation therapy).

Concerning VEGF-A, higher continuous mRNA expression values were associated with higher age (≥50 years, Mann-Whitney test, *P *= 0.001), postmenopausal status (*P *= 0.001), negative ER/PgR status (*P *<0.001), positive HER2 status (*P *= 0.020), higher grade (III-IV, *P *= 0.027) and no adjuvant hormonal therapy (*P *= 0.003). Higher VEGF-B mRNA expression values were associated with higher age (*P *<0.001), postmenopausal status (*P *= 0.002), positive ER/PgR status (*P *= 0.023) and lower grade (I-II, *P *= 0.024). No statistically significant associations were found for VEGFR2, while higher mRNA expression values of VEGFR3 were associated with higher age (*P *= 0.030) (Table 2).

**Table 2:** Association of VEGF-A, VEGF-B, VEGFR2 and VEGFR3 mRNA expression with basic patient and tumor characteristics.

		VEGF-A mRNA expression			VEGF-B mRNA expression			VEGFR2 mRNA expression			VEGFR3 mRNA expression		
		(Number = 307)			(Number = 304)			(Number = 308)			(Number = 308)		
		
		Median	Range	*P *value	Median	Range	*P *value	Median	Range	*P *value	Median	Range	*P *value
Age	<50	34.8	(32.4-38.3)	**0.001**	35.3	(33.1-37.5)	**<0.001**	32.1	(29.6-34.4)	0.190	31.9	(27.4-34.2)	**0.030**
								
	≥50	35.3	(28.2-38.3)		35.7	(27.5-38.0)		32.2	(29.2-34.5)		32.1	(29.1-34.4)	

Treatment group	E-T-CMF	35.0	(33.4-38.3)	0.629	35.5	(27.5-37.4)	0.13	32.1	(29.5-34.5)	0.99	32.0	(27.4-34.0)	0.47
								
	E-CMF	35.1	(28.2-38.3)		35.5	(32.4-38.0)		32.1	(29.2-34.4)		32.1	(29.1-34.4)	

Menopausal status	Premenopausal	34.8	(32.4-38.3)	**0.001**	35.4	(33.1-37.5)	**0.002**	32.1	(29.6-34.4)	0.17	31.9	(27.4-34.2)	0.069
								
	Postmenopausal	35.3	(28.2-38.3)		35.7	(27.5-38.0)		32.3	(29.2-34.5)		32.1	(29.1-34.4)	

ER/PgR status	Negative	35.5	(32.4-38.3)	**<0.001**	35.4	(27.5-38.0)	**0.023**	32.3	(29.6-34.4)	0.310	32.1	(27.4-34.4)	0.288
								
	Positive	34.9	(33.1-38.0)		35.6	(33.4-37.6)		32.1	(29.2-34.5)		32.0	(29.1-34.4)	

HER2 status^a^	Negative	35.0	(28.2-38.2)	**0.020**	35.5	(32.4-37.5)	0.856	32.0	(29.2-34.5)	0.088	32.0	(29.1-34.4)	0.294
								
	Positive	35.4	(33.4-38.3)		35.6	(27.5-38.0)		32.4	(29.5-34.4)		32.4	(27.4-34.4)	

Positive nodes	0 to 3	35.0	(32.4-38.1)	0.97	35.4	(33.7-37.2)	0.51	32.0	(30.3-34.1)	0.42	31.9	(30.0-34.2)	0.35
								
	≥4	35.0	(28.2-38.3)		35.6	(27.5-38.0)		32.1	(29.2-34.5)		32.0	(27.4-34.4)	

Tumor size	≤2	34.9	(28.2-38.0)	0.13	35.7	(33.4-36.9)	0.140	32.2	(29.6-34.3)	0.44	32.2	(27.4-34.4)	0.43
								
	2-5	35.0	(32.4-38.3)		35.4	(27.5-38.0)		32.0	(29.2-34.5)		32.0	(29.1-34.4)	
								
	>5	35.3	(33.1-37.9)		35.5	(33.1-37.6)		32.2	(29.5-34.0)		32.1	(29.4-34.1)	

Histological grade	I-II	34.9	(28.2-38.0)	**0.027**	35.6	(33.1-37.6)	**0.024**	32.2	(29.9-34.4)	0.25	32.1	(29.5-34.4)	0.12
								
	III-IV	35.1	(32.4-38.3)		35.4	(27.5-38.0)		32.0	(29.2-34.5)		32.0	(27.4-34.4)	

Adjuvant HT	No	35.8	(33.1-37.9)	**0.003**	35.5	(33.8-36.8)	0.59	32.1	(30.1-33.9)	0.85	31.8	(29.9-33.6)	0.62
								
	Yes	34.9	(28.2-38.3)		35.5	(27.5-38.0)		32.1	(29.2-34.5)		32.0	(27.4-34.4)	

Adjuvant RT	No	35.4	(32.4-37.9)	0.17	35.4	(33.4-37.2)	0.72	32.0	(30.1-34.1)	0.52	31.9	(29.4-33.9)	0.48
								
	Yes	35.0	(28.2-38.3)		35.5	(27.5-38.0)		32.1	(29.2-34.5)		32.1	(27.4-34.4)	

Normalized mRNA expression values (40-delta CT) are presented. Comparisons were made using the Mann-Whitney test, except for tumor size where the Kruskall-Wallis test was used. ^a^Positive HER2 status: HER2 3+ by IHC and/or *HER2 *amplification by FISH. Significant *P *values are shown in bold. CMF, cyclophosphamide, methotrexate, fluorouracil; CT, cycle threshold; ER, estrogen receptor; FISH, fluorescence *in situ *hybridization; HER2, human epidermal growth factor receptor 2; HT, hormonal therapy; PgR, progesterone receptor; RT, radiation therapy; VEGF, vascular endothelial growth factor; VEGFR, vascular endothelial growth factor receptor.

Associations of VEGF-C and VEGFR1 mRNA status (high versus low at the 75^th ^percentile) with selected clinicopathological factors are shown in Table 3. High mRNA expression of VEGF-C was associated with higher age (≥50 years, Fisher's exact test, *P *= 0.024), while ER/PgR-negative tumors were more frequent in high VEGF-C expressing tumors (37.7% in high versus 17.3% in low, *P *= 0.001). Similarly, HER2-positive tumors were more frequent in high VEGF-C expressing tumors (46.6% in high versus 21.5% in low, *P *<0.001). Overall, high VEGF-C expression was more frequent in ER/PgR-negative and HER2-positive tumors. The number of positive lymph nodes did not seem to be associated with the expression of VEGF-C (*P *= 0.17). Concerning VEGFR1, ER/PgR-negative tumors and HER2-positive tumors were more frequent in high VEGFR1 expressing tumors (33.3% in high versus 18.8% in low, *P *= 0.021 and 39.7% in high versus 23.7% in low, *P *= 0.028, respectively). Finally, high expression of VEGFR1 was associated with adjuvant radiotherapy (*P *= 0.036).

**Table 3:** Association of VEGF-C and VEGFR1 mRNA expression with basic patient and tumor characteristics.

		VEGF-C mRNA expression (Number = 305)			VEGFR1 mRNA expression (Number = 306)		
		
		Low (*n *= 229) Number (%)	High (*n *= 76) Number (%)	*P *value	Low (*n *= 230) Number (%)	High (*n *= 76) Number (%)	*P *value
Age	<50	123 (53.9)	29 (38.2)	**0.024**	118 (51.5)	35 (46.1)	0.43
				
	≥50	105 (46.1)	47 (61.8)		111 (48.5)	41 (53.9)	

Treatment group	E-T-CMF	113 (49.3)	28 (36.8)	0.064	110 (47.8)	30 (39.5)	0.23
				
	E-CMF	116 (50.7)	48 (63.2)		120 (52.2)	46 (60.5)	

Menopausal status	Premenopausal	130 (56.8)	34 (44.7)	0.084	128 (55.7)	37 (48.7)	0.35
				
	Postmenopausal	99 (43.2)	42 (55.3)		102 (44.3)	39 (51.3)	

ER/PgR status	Negative	34 (17.3)	23 (37.7)	**0.001**	37 (18.8)	20 (33.3)	**0.021**
				
	Positive	162 (82.7)	38 (62.3)		160 (81.2)	40 (66.7)	

HER2 Status^a^	Negative	146 (78.5)	31 (53.5)	** <0.001**	142 (76.3)	35 (60.3)	**0.028**
				
	Positive	40 (21.5)	27 (46.6)		44 (23.7)	23 (39.7)	

Positive nodes	0-3	61 (26.6)	14 (18.4)	0.17	61 (26.5)	14 (18.4)	0.17
				
	≥4	168 (73.4)	62 (81.6)		169 (73.5)	62 (81.6)	

Tumor size	≤2	61 (26.6)	30 (39.5)	0.093	64 (27.8)	27 (35.5)	0.40
				
	2-5	127 (55.5)	33 (43.4)		123 (53.5)	38 (50.0)	
				
	>5	41 (17.9)	13 (17.1)		43 (18.7)	11 (14.5)	

Histological grade	I-II	113 (49.3)	41 (53.9)	0.51	118 (51.3)	37 (48.7)	0.79
				
	III-IV	116 (50.7)	35 (46.1)		112 (48.7)	39 (51.3)	

Adjuvant HT	No	17 (7.4)	9 (11.8)	0.24	20 (8.7)	6 (7.9)	0.99
				
	Yes	212 (92.6)	67 (88.2)		210 (91.3)	70 (92.1)	

Adjuvant RT	No	43 (18.9)	11 (14.5)	0.49	47 (20.5)	7 (9.3)	**0.036**
				
	Yes	184 (81.1)	65 (85.5)		182 (79.5)	68 (90.7)	

Cut-off values were set at the 75th percentile of the marker's distribution. Comparisons were made using the Fisher's exact test. ^a^Positive HER2 status: HER2 3+ by IHC and/or *HER2 *amplification by FISH. Significant *P *values are shown in bold. CMF, cyclophosphamide, methotrexate, fluorouracil; ER, estrogen receptor; FISH, fluorescence *in situ *hybridization; HER2, human epidermal growth factor receptor 2; HT, hormonal therapy; PgR, progesterone receptor; RT, radiation therapy; VEGF, vascular endothelial growth factor; VEGFR, vascular endothelial growth factor receptor.

### Association of vascular endothelial growth factor ligands with survival

#### VEGF-A and VEGF-B

VEGF-A and VEGF-B mRNA values did not achieve prognostic significance in any of the distribution cut-offs examined. Cox regression analysis, adjusted for treatment group, for the continuous normalized mRNA expression values of VEGF-A failed to establish a distinct risk for death (HR = 1.14, 95% CI: 0.94 to 1.39, Wald's *P *= 0.18) or risk for relapse (HR = 1.09, 95% CI: 0.92 to 1.29, *P *= 0.30). Similarly, normalized mRNA expression values of VEGF-B did not have prognostic significance for OS (HR = 0.86, 95% CI: 0.71 to 1.05, Wald's *P *= 0.14) or DFS (HR = 0.93, 95% CI: 0.77 to 1.14, *P *= 0.50) when analyzed as a continuous variable.

Patients were randomized to a taxane-free versus a taxane-containing chemotherapy and, thus, the predictive significance of VEGF markers for the paclitaxel-containing adjuvant chemotherapy treatment was examined as well. There was no significant interaction between VEGF-A and VEGF-B with chemotherapy treatment in terms of OS or DFS (tests for interaction, Wald's *P *>0.062 in all cases).

#### VEGF-C

**Figure 2:**
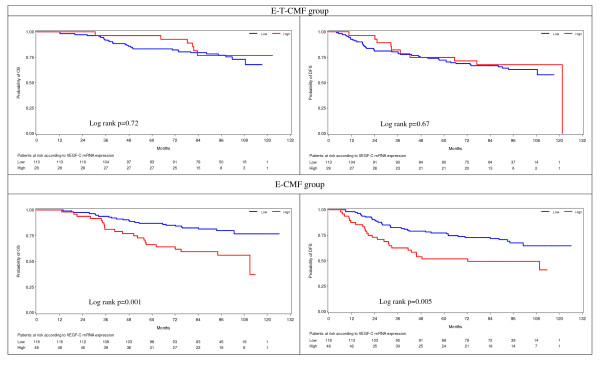
**Kaplan-Meier curves according to VEGF-C mRNA expression and treatment**. OS and DFS for patients with low VEGF-C mRNA expression (blue line) and high VEGF-C mRNA expression (red line) randomized in the E-T-CMF and E-CMF treatment groups. Interaction between VEGF-C mRNA expression and treatment group was significant for OS (*P *= 0.019) and DFS (*P *= 0.041). CMF, cyclophosphamide, methotrexate, fluorouracil; DFS, disease-free survival; OS, overall survival; VEGF-C, vascular endothelial growth factor C.

The cut-off for VEGF-C was set at the 75^th ^percentile of the marker's distribution. VEGF-C was predictive for benefit from adjuvant treatment with paclitaxel (E-T-CMF arm) for OS (test for interaction, Wald's *P *= 0.038), and marginally significant for DFS (test for interaction, Wald's *P *= 0.055). The impact of VEGF-C expression on OS and DFS in the two treatment groups is shown in Figure 2. Patients with high VEGF-C mRNA expression randomized to the non paclitaxel-containing adjuvant chemotherapy arm (E-CMF) had decreased OS and DFS (log-rank, *P *= 0.001 and *P *= 0.005, respectively; HR for OS = 2.57, 95% CI: 1.42 to 4.65; HR for DFS = 2.10, 95% CI: 1.26 to 3.51) compared to the patients with low VEGF-C expression, while no difference in OS or DFS was detected in the E-T-CMF group (log-rank, *P *= 0.72 and *P *= 0.67, respectively; HR for OS = 0.84, 95% CI: 0.35 to 2.02 for high VEGF-C expression; HR for DFS = 0.89, 95% CI: 0.44 to 1.80 for high VEGF-C expression).

### Association of vascular endothelial growth factor receptors with survival

**Figure 3:**
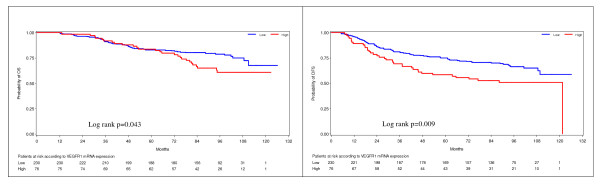
**Kaplan-Meier curves according to VEGFR1 mRNA expression**. High mRNA expression of VEGFR1 (above the 75^th ^percentile) was associated with significantly reduced OS (left) and DFS (right). DFS, disease-free survival; OS, overall survival; VEGFR1, vascular endothelial growth factor receptor 1.

For the VEGFR1 receptor the 75^th ^percentile was prognostic for both OS and DFS, while for the VEGFR2 and VEGFR3 receptors no prognostic significance was found in the examined cut-offs in terms of OS or DFS. Concerning VEGFR1, 27/76 deaths (36%) and 37/76 relapses (49%) occurred in patients with high expressing tumors, in comparison to 52/230 deaths (23%) and 76/230 relapses (33%) in the low expressing tumors. Moreover, patients with high mRNA expression of VEGFR1 had increased risk for death (HR = 1.60, 95% CI: 1.01 to 2.55, Wald's *P *= 0.047) and increased risk for relapse (HR = 1.67, 95% CI: 1.13 to 2.48, *P *= 0.010) compared to patients with low expressing tumors when adjusting for treatment group. Kaplan-Meier curves for OS and DFS according to the mRNA status of VEGFR1 are presented in Figure 3.

Examining continuous normalized mRNA expression values of VEGFR2, Cox regression analysis, adjusted for treatment group, did not show any associations with OS (HR = 1.10, 95% CI: 0.88 to 1.38, Wald's *P *= 0.41), or DFS (HR = 1.12, 95% CI: 0.93 to 1.35, *P *= 0.23). Similarly, normalized mRNA values of VEGFR3 did not have prognostic significance for OS (HR = 0.97, 95% CI: 0.78 to 1.19, Wald's *P *= 0.75), or DFS (HR = 1.07, 95% CI: 0.89 to 1.28, *P *= 0.47) when analyzed as a continuous variable.

Regarding predictive ability, there were no significant interactions between VEGF receptors and adjuvant chemotherapy for either OS or DFS (Wald's *P *>0.24 in all cases).

### Interactions between ligands and receptors

Interactions between all possible combinations of ligands and receptors (VEGF-A*VEGFR1, VEGF-A*VEGFR2, VEGF-B*VEGFR1, VEGF-C*VEGFR2, VEGF-C*VEGFR3) were tested, both for OS and DFS. The interaction between VEGF-A and VEGFR1, adjusted for treatment group, was found to be significant in terms of OS (Wald's *P *= 0.017). More specifically, for those patients with low expression of VEGFR1 a one unit rise of the VEGF-A mRNA expression value would lead to increased risk for death with an HR of 1.43 (95% CI: 1.11 to 1.83), whereas for patients with high expression of VEGFR1 a one unit rise of the VEGF-A mRNA expression value would lead to an HR for OS of 0.84 (95% CI: 0.59 to 1.20).

### Multivariate Cox regression model for overall survival and disease-free survival adjusting for clinical parameters

The Cox multivariate regression analysis for OS (Table 4) revealed that the hazard of death at any time was significantly higher for patients with more than three positive nodes (HR = 2.58, 95% CI: 1.32 to 5.02, Wald's *P *= 0.005), higher histological grade (HR = 1.94, 95% CI: 1.21 to 3.11, *P *= 0.006) and no hormonal therapy (HR = 2.86, 95% CI: 1.56 to 5.26, *P *= 0.001). Among the VEGF family members evaluated, VEGF-B and VEGF-C were associated with risk for death. For a one-unit increase in the mRNA expression of VEGF-B there was an 18% decrease in risk for death (*P *= 0.019). There was also a statistically significant difference in the treatment effect according to VEGF-C expression (*P *for interaction 0.019). The same clinicopathological factors had significant prognostic value for DFS: high histological grade (III-IV, *P *= 0.002), four or more positive nodes (*P *<0.001) and adjuvant hormonal therapy (*P *= 0.008), while VEGF-B (*P *= 0.084), VEGFR1 (*P *= 0.060) and the change in treatment effect on the hazard for disease progression according to VEGF-C mRNA expression were also statistically significant (*P *for interaction 0.041). Overall, there was a decreased, but not significant, risk for death in tumors with high VEGF-C expression (HR = 0.74, 95% CI: 0.28 to 1.96, *P *= 0.547), as well as a non-significant decreased risk for relapse (HR = 0.68, 95% CI: 0.31 to 1.48, *P *= 0.327) in the E-T-CMF group. Regarding the E-CMF group, high expression of VEGF-C increased the risk for death (HR = 2.85, 95% CI: 1.55 to 5.22, Wald's *P *<0.001) and the risk for relapse (HR = 1.73, 95% CI: 0.98 to 3.08, *P *= 0.166).

**Table 4:** Multivariate analysis for prognostic significance: parameters in the final Cox model.

Overall survival	HR	95% CI	Wald's *P*
Histological grade			
III-IV versus I-II	1.94	1.21-3.11	0.006
Number of positive nodes			
≥4 versus 0 to 3	2.58	1.32-5.02	0.005
Adjuvant HT			
No versus Yes	2.86	1.56-5.26	0.001
VEGF-B			
Continuous mRNA values	0.82	0.69-0.97	0.019
			
VEGF-C/Treatment group Interaction	3.84	1.24-11.84	0.019
Treatment group			
E-CMF versus E-T-CMF for VEGF-C low	0.96	0.54-1.70	0.885
E-CMF versus E-T-CMF for VEGF-C high	3.68	1.38-9.80	0.009
VEGF-C			
High versus Low for E-T-CMF	0.74	0.28-1.96	0.547
High versus Low for E-CMF	2.85	1.55-5.22	<0.001

**Disease-free survival**	**HR**	**95% CI**	**Wald's *P***

Histological grade			
III-IV versus I-II	1.83	1.24-2.71	0.002
Number of positive nodes			
≥4 versus 0 to 3	2.80	1.58-4.95	<0.001
Adjuvant HT			
No versus Yes	2.16	1.22-3.84	0.008
VEGF-B			
Continuous mRNA values	0.86	0.72-1.02	0.084
VEGFR1			
High versus Low	1.58	0.98-2.55	0.060
			
VEGF-C/Treatment group Interaction	2.56	1.04-6.31	0.041
Treatment group			
E-CMF versus E-T-CMF for VEGF-C low	0.95	0.60-1.52	0.846
E-CMF versus E-T-CMF for VEGF-C high	2.44	1.12-5.31	0.057
VEGF-C			
High versus Low for E-T-CMF	0.68	0.31-1.48	0.327
High versus Low for E-CMF	1.73	0.98-3.08	0.166

CI, confidence interval; CMF, cyclophosphamide, methotrexate, fluorouracil; HR, hazard ratio; HT, hormonal therapy; VEGF, vascular endothelial growth factor.

## Discussion

Experimental and clinical evidence is rapidly accumulating regarding the significant role of angiogenesis in breast cancer progression and metastasis. VEGF has emerged as possibly the most essential angiogenic factor, expressed in many tumors including breast cancer, where it has been investigated for more than a decade now for its prognostic significance [35]. In most studies, VEGF expression is measured by IHC [36] or ELISA [37], but recently, PCR-based methods have also been used to assess VEGF mRNA expression in tumor tissues [38]. In general, PCR-based methods have proven to be very effective for the quantitative analysis of gene copy number or mRNA, especially when only a limited amount of tissue is available [39,40], while recent publications have shown that total RNA isolated from FFPE tissue samples can be used for reliable gene expression analysis [22,41]. Furthermore, Oncotype DX is a clinically validated prognostic test for patients with breast cancer, based on a qRT-PCR multigene algorithm [42]. It is worth noting that Oncotype DX does not include angiogenesis markers, but rather proliferation genes and other known prognostic genes, such as *ER *and *HER2*; therefore, the identification of useful prognostic indicators among the VEGF family members could have potential applications in similar multigene platforms. Furthermore, evidence is lacking on the ability of VEGF family members to predict benefit from specific treatments, especially on their predictive value for bevacizumab use. Several interesting candidate biomarkers for anti-angiogenic therapies have been evaluated in recent translational research studies and many are currently under investigation in prospective clinical trials. A recent report has shed some light on this issue by exploring biomarkers of the VEGF family for their possible effect on bevacizumab [43]. Results were only indicative that patients with low VEGF-C, among other markers, show trends toward improvement in progression-free survival associated with the addition of bevacizumab to capecitabine. Also, in a recently published biomarker evaluation study from the AVAGAST randomized trial in advanced gastric cancer, plasma VEGF-A and neuropilin-1 emerged as potential predictors of bevacizumab response [44].

In the present study, we analyzed the mRNA expression of well-recognized VEGF family members, including receptors (VEGFR1, 2 and 3) and their ligands (VEGF-A, B and C) in an attempt to identify individual members with prognostic/predictive significance. Our patient cohort included early breast cancer patients with high-risk characteristics: half were premenopausal, the majority had ≥4 positive axillary lymph nodes, large tumor size in most cases, almost half had high grade tumors, while 18.5% had ER/PgR-negative and 21.8% HER2-positive tumors. These patients participated in an adjuvant clinical study and were randomized to receive anthracycline-based chemotherapy with or without a taxane (E-T-CMF versus E-CMF). In this high-risk population, increased levels of VEGF-A mRNA were significantly associated with certain negative prognostic indicators, such as negative ER/PgR status, higher histological grade, positive HER2 status and no adjuvant hormonal therapy. VEGF-A mRNA levels have previously been associated with breast tumor characteristics, such as histological type and grade, albeit with variable results [45,46].

The prognostic value of VEGF family members on survival has been assessed in our patient population. Neither VEGF-A nor -B had prognostic significance for OS; they had no significant interaction with the chemotherapy treatment arm. With regard to receptors, only high expression of VEGFR1 was prognostic for both OS and DFS.

The prognostic value of VEGF-A expression has been assessed by IHC in several studies [15,19,37]. Recent retrospective clinical studies have strengthened the prognostic significance of total VEGF, as assessed by IHC in breast cancer [47,48], and have resulted in the recognition of the importance of VEGF as a possibly predictive biomarker and target for therapy in the more aggressive subcategory of triple-negative breast cancer [49,50]. Recent clinical evidence also strengthens the need for anti-angiogenic treatment in the triple-negative subtype, as bevacizumab added to neoadjuvant chemotherapy significantly increased the pathological complete response among patients with HER-negative early breast cancer, and primarily, those with triple-negative tumors [51]. However, in all of the above-mentioned studies, VEGF expression was assessed with standard IHC methods only [15,19,37,47-51]. In our study population, total VEGF had previously been assessed by IHC together with HER2, and, while HER2 was a negative prognostic indicator, high VEGF protein expression was not significantly associated with either DFS or OS [52].

It is important to note that, in our patient cohort, high mRNA expression of VEGFR1 had prognostic significance and, furthermore, the interaction of VEGF-A with VEGFR1 showed prognostic significance as well, while high expression of the ligand alone did not. This underlines the possible importance of interactions within the VEGF family, rather than that of individual members, and strengthens the need for further investigation. The binding of multiple ligands to individual receptors has previously been described [39]; however, certain interactions appear to be more important than others. According to the findings of our study, the correlation of VEGF-A with tumor profile, namely that higher expression was to be expected when the tumor was more aggressive, is not reflected by a negative prognostic effect on OS or DFS. There is, however, evidence of a negative prognostic role of increased VEGF-A mRNA expression in the low VEGFR1 subgroup with respect to OS. This particular subgroup of patients has a more favorable tumor profile in terms of ER/PgR and HER2 than the subgroup with high VEGFR1 levels. Therefore, a larger study should be conducted to explore whether the strong effect on DFS/OS exhibited by the receptor (VEGFR1) is masking the possible prognostic value of the VEGF-A ligand.

The most significant findings in our study involved VEGF-C; this factor emerged as a very important member of the VEGF family. In agreement with recent evidence from a number of studies, associations were found with VEGF-C and aggressive phenotype characteristics; ER/PgR-negative tumors and HER2-positive tumors had high VEGF-C expression more frequently. It is known that VEGF-C is a potent enhancer of tumor lymphangiogenesis, leading to increased metastatic spread of breast cancer cells to lymph nodes; however, in our study no significant correlation was found between the level of VEGF-C mRNA expression (low/high) and the number of positive lymph nodes (0 to 3 versus ≥4). However, it needs to be noted that the vast majority of patients in our study had large numbers of positive lymph nodes (>75% of the patients had ≥4 positive axillary lymph nodes); therefore, conclusive correlations were not possible. In a previous study, a significant association between increased VEGF-C expression and advanced histological grade was found, suggesting that poorly differentiated tumor cells may be more capable of secreting VEGF-C, which can induce lymphangiogenesis in breast cancer [47], while VEGF-C together with extracellular matrix protein 1 were found overexpressed in breast cancer lymphatic metastases [53]. It is also important to note that in our study, high VEGF-C and VEGFR1 mRNA expression was more frequently seen in HER2-positive tumors, indicating that certain VEGF family members could prove to be even more useful when analyzed in combination with other markers, with potential, for instance, to recognize patients with poor prognosis among the HER2-positive or, more importantly, the HER2-negative populations.

An important finding in our study was the predictive significance of VEGF-C and the impact of the taxane-containing treatment arm. Patients with high VEGF-C expressing tumors benefited more from the addition of paclitaxel in terms of OS. This was also evident in the multivariate analysis: patients with high VEGF-C mRNA expression were those with the worse prognosis, and they appear to benefit more from the taxane-containing treatment, possibly through the potential anti-angiogenic properties of the taxane therapy. Weekly taxane administration is considered very effective, both in the neoadjuvant and metastatic settings [54,55] and recently in the adjuvant setting [56]. Furthermore, there is evidence for anti-angiogenic effects of this schedule in addition to the anti-microtubule properties [57]. In our study, taxane treatment was indeed delivered in a dose-dense manner, every two weeks. The interaction of VEGF-C expression with treatment provides a significant indication for a possible predictive role of mRNA expression of VEGF family members, a role that warrants further evaluation in larger studies.

In our study, VEGF family mRNA expression and, in particular, high VEGF-C and VEGFR1 expression, was able to identify those patients with early breast cancer who have a higher likelihood of recurrence or death than those with low-angiogenic tumors, even if treated with adjuvant chemo-hormonotherapy. The taxane-containing treatment administered in a dose-dense manner, might have offered anti-angiogenic effects, which seem to be of more benefit for those patients with a high expression of angiogenic markers, such as VEGF-C and VEGFR1. The high expression of these factors might reflect subcategories of high-angiogenic tumors. It may be that such patient subsets represent good candidates for testing additional strategies to complement chemotherapy, such as anti-VEGF targeting agents in combination with conventional therapies. The results of our study provide the first evidence toward the identification of relevant angiogenic biomarkers in dose-dense chemotherapy regimens. Recent evidence of the strong predictive value of VEGF in premenopausal early breast cancer patients [58], as well as the predictive significance of tumor angiogenesis in high-risk early breast cancer patients [59], underlines the need for additional studies that could possibly support and/or clarify these findings.

## Conclusions

In conclusion, the present study reports, for the first time, that VEGF-C mRNA overexpression, as assessed by qRT-PCR, has a strong predictive value in high-risk early breast cancer patients undergoing adjuvant dose-dense taxane-containing chemotherapy. Further studies are warranted to validate the prognostic and/or predictive value of VEGF-B, VEGF-C and VEGFR1 in patients treated with adjuvant therapies and to reveal which members of the VEGF family might possibly be useful in identifying those patients who will benefit most from anti-VEGF strategies.

## Abbreviations

bp: base pair; CI: confidence interval; CMF: cyclophosphamide: methotrexate: fluorouracil; CT: cycle threshold; DFS: disease-free survival; E: epirubicin; ELISA: enzyme-linked immunosorbent assay; ER: estrogen receptor; FFPE: formalin-fixed paraffin-embedded; Flt-1: Fms-related tyrosine kinase 1; Flt-4: Fms-related tyrosine kinase 4; FISH: fluorescence *in situ *hybridization; G-CSF: granulocyte-colony stimulating factor; H & E: hematoxylin and eosin; HER2: human epidermal growth factor receptor 2; HT: hormonal therapy; HeCOG: Hellenic Cooperative Oncology Group; HR: hazard ratio; IHC: immunohistochemistry; KDR/Flk-1: kinase insert domain receptor/fetal liver kinase; OS: overall survival; PgR: progesterone receptor; qRT-PCR: quantitative reverse transcriptase-polymerase chain reaction; RT: radiation therapy; RT-PCR: reverse transcriptase-polymerase chain reaction; T: taxol (Paclitaxel); TMA: tissue microarray; VEGF (A: B: C): vascular endothelial growth factor (A: B: C); VEGFR (1: 2: 3): vascular endothelial growth factor receptor (1: 2: 3); ΔCT: delta cycle threshold.

## Competing interests

On behalf of the Hellenic Foundation for Cancer Research, Athens, Greece, the senior author (GF) has pending patent applications with Siemens Healthcare Diagnostics, Tarrytown, NY. The rest of the authors declare that they have no competing interests.

## Authors' contributions

HL conceived of the study, participated in its design and coordination and drafted the manuscript. KTK conceived of the study, participated in its design and coordination and drafted the manuscript. RK carried out the molecular studies and helped to draft the manuscript. GK participated in the design of the study and performed the statistical analysis. RMW carried out the molecular studies and helped to draft the manuscript. FZ participated in the clinical management of the patients and contributed to the collection of the tumor tissue samples analyzed in the study. HG, CC, AKK, ES, DP and DB participated in the clinical management of the patients and contributed to the collection of the tumor tissue samples analyzed in the study. GF conceived of the study, participated in its design and coordination and helped to draft the manuscript. All authors read and approved the final manuscript.

## Authors' information

Current address for Dr. Ralf Kronenwett: Sividon Diagnostics GmbH, Nattermann Allee 1, D-50829 Cologne, Germany.

Current address for Dr. Ralph M. Wirtz: Stratifyer Molecular Pathology GmbH, Werthmannstrasse 1, D-50935 Cologne, Germany.
